# Study methodology in trauma care: towards question-based study designs

**DOI:** 10.1007/s00068-019-01248-5

**Published:** 2019-10-29

**Authors:** Roderick M. Houwert, Reinier B. Beks, Marcel G. W. Dijkgraaf, Kit C. B. Roes, F. Cumhur Öner, Falco Hietbrink, Luke P. H. Leenen, Rolf H. H. Groenwold

**Affiliations:** 1 Department of Surgery, University Medical Center Utrecht, Utrecht, The Netherlands; 2 Department of Surgery, Northwest Clinics Alkmaar, Wilhelminalaan 12, 1815JD Alkmaar, The Netherlands; 3 Department of Clinical Epidemiology, Biostatistics and Bioinformatics, Amsterdam UMC, University of Amsterdam, Amsterdam, The Netherlands; 4 Department of Biostatistics, Julius Center, University Medical Centre Utrecht, Utrecht, The Netherlands; 5 Department of Orthopedic Surgery, University Medical Center Utrecht, Utrecht, The Netherlands; 6 Department of Clinical Epidemiology, Leiden University Medical Centre, Leiden, The Netherlands

## Abstract

The randomized controlled trial (RCT) in surgery may not always be ethical, feasible, or necessary to address a particular research question about the effect of a surgical intervention. If so, properly designed and conducted observational (non-randomized) studies may be valuable alternatives for an RCT and produce credible results. In this paper, we discus differences between RCTs and observational studies and differentiate between three types of comparisons of surgical interventions. We assert that results of different designs should be regarded as complementary to each other when evaluating surgical interventions. Criteria for credible observational research are presented to provide guidance for future observational research of surgical interventions. We argue that the research question that is being asked should guide the discussion about the value of a particular study design.

## Introduction

Publication of the streptomycin drug trial in 1948 started the widespread adoption of the randomized controlled trial (RCT) design for pharmacological interventions [1]. Due to its theoretical advantage of eliminating all confounding factors the RCT has become the gold standard for the assessment of different interventions, whereas results from non-randomized or observational studies are thought to be less reliable because of potential bias [2]. Hence, several initiatives have been engaged to establish guidelines and formats in support of a more widespread application of surgical RCTs [3]. These initiatives are substantiated by examples from the literature where surgical interventions have been discontinued after results from large RCTs contradicted results from observational studies [4, 5]. RCTs are indeed increasingly applied nowadays, but observational studies continue to account for the majority of the published research [6]. RCTs in surgery may not always be ethical, feasible, or necessary to address a particular research question. If so, properly designed and conducted observational studies can be valuable alternatives for an RCT and produce credible results [7–11]. In this paper, we elaborate on complementary use of observational studies and RCTs and the need for both.

## Difficulties of RCTs in surgical research

From a methodological perspective, surgical research is a different entity compared to pharmacological research. Surgical RCTs have additional challenges and obstacles to overcome [6, 12]. The working mechanism of medical drugs and their administration are rather 'abstract' for a patient, while surgical treatment is a tangible and usually highly invasive concept. Consequently patients may have a stronger treatment preference and are less willing to participate in randomization between different surgical procedures [13]. Another obstacle is the preference of the surgeon for a specific surgical treatment as a result of technical skills, personal experience, professional culture, and infrastructure at participating centers [14]. Consequently, equipoise at the level of the treating surgeon needed to perform an RCT, is difficult to achieve. In light of these concerns, the use of expertise-based randomised trials has been proposed as an alternative design, where participants are randomized to surgeons with expertise in the allocated intervention [15]. However, an expertise-based trial may not necessarily produce a result that is generalisable to the whole surgical community, due to the make-up of the participating surgeons and centers [16].

What is more, surgical techniques evolve parallel to treatment due to the necessary learning curve and further development of the surgical procedure. This phenomenon hampers direct comparison with the gold standard in an RCT during the early development phase.

Further, surgery as opposed to pharmacological treatment is a combination of pre-, peri-, and postoperative procedures subject to the skills and expertise of the surgical team as well as to the logistics and infrastructure of the hospital (e.g. operating room availability, intensive care facility, supporting specialities). In fact, surgery is a multifaceted process and hence constitutes a “complex intervention”. It is inherently difficult to assess the added value of each distinct element in the chain by experimental design.

Finally, on the very practical end of the spectrum, surgical RCTs often lack the funding usually associated with evaluating new drugs or new medical devices.

These challenges are especially pronounced in the surgical field with acute and urgent life-threatening situations, such as trauma surgery, where variation in surgical practice can lead to practical and methodological difficulties in terms of patient recruitment and randomization.

## Comparison of study designs for intervention studies

Given the challenges and obstacles to perform a surgical RCT compared to an observational study it is important to understand to what extent differences between these designs impact study results. Concato et al., Benson et al., and Ioannidis et al. provide an empirical basis for a comparison between different study designs [17–19]. They reviewed the literature about various medical interventions and compared results of trials to those obtained from observational research about the same interventions. The majority of the topics they considered included pharmacological treatments, but studies of surgical intervention were included as well. Based on their reviews, one could get the impression that results from the different designs are rather similar (Concato and Benson), but also that results can be quite different (Ioannidis). Shikata et al. performed a similar comparison between research design, but restricted the comparison to 18 digestive surgery topics and found a significant difference between different study designs (observational vs RCT) in 25% of the primary outcomes assessed [20]. Similar findings were presented more recently by Edwards et al. for breast cancer surgery; they found differences in study results between designs in 2 out of 10 topics [21]. The overall conclusion, based on theoretical rather than empirical considerations, seems to be that RCTs have superior validity (notably due to the randomization), yet in certain situations the different designs may yield actually quite similar results. As earlier described by Kunz et al., the unpredictability of bias is the main problem of observational studies [22]. If the exact size and direction of bias is known, it would be easy to approximate "true" results by subtracting the effect of bias from the study result. An obvious question that emerges is what are the drivers of difference between the different designs and in which situation might an observational study design be considered appropriate as well? It may depend on the research question that is being asked.

## Different research questions in surgical intervention studies

A key difference between RCTs and observational studies is the potential for confounding in the latter due to the absence of randomization. In observational research of a medical treatment effect, allocation of treatment follows clinical practice and therefore treated and untreated patients often differ on prognostic characteristics [23]. When comparing groups of treated and untreated patients, such incomparability almost always leads to bias, known as confounding. Particularly the potential for unobserved confounding is considered an important reason for differences in the effects obtained from RCTs and effects obtained from observational studies [24]. The potential for confounding likely depends on the type of intervention that is studied and the comparison that is made. In intervention studies in (trauma) surgery, three types of comparisons can be distinguished [6, 25].

### Type 1: Comparison of pharmacological interventions in surgical patients

A type 1 comparison focuses on pharmacological interventions in surgical patients, comparing a particular drug with a placebo or active comparator (other drug). The focus of this kind of research is to quantify the effects of a particular compound (efficacy) or a particular treatment strategy (effectiveness). Efficacy is the effect of an intervention under controlled circumstances, whereas effectiveness refers to the effect of an intervention in daily clinical practice [26]. In daily practice, prescriptions of pharmacological interventions often are based on a clinical indication for that drug. Patient characteristics and severity of disease are important factors in decision-making (e.g. about initiating treatment) and consequently who ends up in which treatment group. Observational studies of pharmacological interventions are therefore generally highly susceptible to confounding, which in this context is sometimes referred to as confounding by indication. We showed this phenomenon in a recent meta-analysis where the pooled therapeutic effect size of vancomycin powder was directly related to the quality of observational studies and the quality of confounding control [27]. Consequently, it is very challenging to study a type 1 comparison using an observational study design and randomization is usually considered indispensable.

### Type 2: Comparison of different surgical interventions

Type 2 comparisons aim at comparing two surgical interventions. A particular operative procedure is compared to a control group that undergoes an alternative operative intervention or a sham operation. Although a type 2 comparison in trauma surgery will generally involve a surgical procedure, type 2 comparisons might focus on an single aspect of the treatment strategy such as follow-up treatment after the surgical procedure; e.g. cast immobilization or unprotected weight-bearing after operative treatment of ankle fractures [28].

Generally, this type of research aims to study the effectiveness of an (operative) procedure as it encompasses a course of pre-, peri-, and postoperative treatment and the treatment effects are subject to skills and expertise of the surgical team; treatment strategies, from admission to discharge, are optimized to the hospital and can differ from other hospitals. Surveys amongst surgeons suggest that for common fracture types different treatment strategies exist due to surgeon preference [14, 29]. Consequently, when two surgical treatment options are considered, skills and expertise of the surgical team will be the most important factor used in decision making. Confounding by indication at the level of the surgical team is thus far less likely to occur. Although referral bias is a common problem in clinical research on elective surgical procedures, patient referral to different surgeons in the trauma and emergency setting is to a large extent a random process. Compared to type 1 research, a comparison of operative interventions is therefore less sensitive to confounding if there are no indications that the surgeon's treatment preference is strongly subject to patient characteristics, and if ‘allocation to surgeon’ is a rather random process [29, 30]. In that case, one can speculate that patient groups who underwent different surgical interventions will be rather similar and therefore results from observational studies will be less prone to confounding. Indeed, in a recent meta-analysis of RCTs and observational studies comparing plate fixation with intramedullary fixation for midshaft clavicle fractures, treatment groups in observational studies were surprisingly similar and consequently effect estimates for the primary and secondary outcome measures were comparable between observational studies and randomized trials [10]. One should keep in mind that referral not only means which hospital is selected for a patient with a given indication, it also includes the question whether or not a patient requires surgery at all. A comparison between two hospitals can be biased, if the two hospitals work under different circumstances (e.g. urban versus rural trauma care) and therefore see a different spectrum of disease severity. Thus, observational type 2 comparisons are less valid, if diagnosis and indication for surgery are difficult and diffuse due to high disease variability (e.g. in spine surgery).

### Type 3: Comparison of surgical and non-surgical intervention

In type 3 comparisons, an operative intervention is compared to a nonoperative intervention, for example surgery versus conservative treatment in patients with a particular fracture. Similar to type 2 comparisons, the focus is on the effectiveness of the treatment. Again surgeon’s preference is an important factor in decision-making. However, type 3 comparisons may have a greater risk of confounding because patient characteristics may have a greater influence on the decision between an operation or conservative treatment than for example the decision between two different operative techniques. Still, for many fracture types there is discussion on whether to operate or not and one could argue that decision making for trauma is based on surgeon preferences or hospital guidelines rather than patient characteristics; depending on hospital expertise, available facilities and the surgical team, treatment can be different between different hospitals [31]. For certain type 3 comparisons an observational study design may therefore be suitable. Similar to type 2 comparisons, multiple recent meta-analyses where surgery was compared to conservative treatment indeed found that treatment groups appeared to be similar and effect estimates were comparable between observational studies and randomized trials [7–9]. In studies of type 3 comparisons, treatment groups may indeed be comparable if the surgeon’s preference for one treatment option over the other is of greater influence on treatment decisions than (unmeasured) patient characteristics.

An example of a study of a type 3 comparisons is the Opvent study, which is a multicenter prospective cohort study, including patients with three or more rib fractures admitted to one of the five participating centers [32]. In two centers, rib fixation is the standard-of-care for flail chest or multiple rib fractures, while in three other centers nonoperative treatment is. Propensity score matching will be used to control for potential confounding of the relation between treatment modality and outcome.

## Combining information of different study designs

Meta-analysis is a valuable tool to assess differences in treatment effects. An increasing number of meta-analyses in trauma topics are published that include results from randomized trials as well as observational studies. Provided observational studies are of high quality, adding information from observational studies to meta-analyses will increase the number of patients available for analysis and can lead to more precise effects estimates, possibilities for subgroup analysis, and may provide more insight in rare and long-term outcomes [7]. Obviously, only high quality observational studies should be included for meta-analysis, or at least sensitivity analysis, stratified by study quality, needs to be conducted [7–9]. Alternatively, statistical methodology has been developed to include external (observational) data in the analysis of randomized trials, that accounts for their (dis-)similarity [33, 34]. Another option could be to perform a meta-analysis while accounting for study quality [35].

## Alternative study designs

In addition to the conventional two-arm randomized trials and the prospective cohort study, several other study designs are available for comparison of different treatment modalities. The first example is the pre-post design, which was applied in two recent studies on the introduction of 24/7 availability of trauma surgeons in Dutch level 1 trauma centers [36]. Both studies showed improvement of trauma care in the post intervention period compared to the pre-intervention period, however in one study mortality rates decreased over time, while in the other only an improvement in process related outcomes was found. The baseline level of care apparently highly influences possible study results: “from rock bottom there is only one way.”

Another example is the prospective parallel study design [37]. In this design, two hospitals, or schools, are compared. In spine surgery, a study investigating traumatic thoracic and lumbar spinal fractures nicely illustrates this concept. Eligibility was retrospectively assessed for each case by a panel of orthopaedic surgeons who were representative of the two medical centers, and who were blinded to the treatment actually administered. Patients were included in the study when there was disagreement on the suggested treatment method. Thus, 2 comparable groups were identified undergoing nonoperative or operative treatment.

## Criteria for credible observational studies (SPACE-ME)

We have discussed several factors that potentially hamper surgical RCTs and distinguished between different surgical research questions. These differ in the extent to which observational studies may provide credible answers. In order to assess the credibility of surgical observational studies, sufficient details about the study need to be reported. Several initiatives aimed at improving the reporting of observational studies of which the STROCSS (Strengthening The Reporting Of Cohort Studies in Surgery) guidelines are the most relevant for surgical studies [38]. Although we encourage surgeons to follow the STROCSS criteria, most are about reporting study results and some major elements with regard to methodological quality are lacking. We propose seven criteria to be reported in order to improve the assessment of the methodological quality of surgical observational research, which are summarized in Table 1.

**Table 1:** Criteria for credible surgical observational studies (SPACE-ME)

Schools: Comparison of established surgical schools Protocol: The study should be registered and a protocol should be published Allocation: Allocation of the intervention can be assumed nearly random Confounding: Possible confounding is measured and accounted for in the analysis Effect size: Anticipated large effect size justifies observational study design	
Multidisciplinary research team: Experienced methodologists and statisticians should be involved Endpoint: Primary outcome should be directly related to the surgical intervention or measured by blind assessors	

Similar to an RCT, an observational study evaluating treatment effects should be registered and a protocol should be published before the start of the study. This ensures correct (prospective) data collection and requires clarification in case of protocol deviation, which will help generate high-quality data and awareness of study limitations. Interventions could be evaluated by comparison of different surgical schools. That is, different treatment strategies exist either between two surgeons (e.g. education), between hospitals (e.g. policy), between regions or international differences (e.g. culture). This gives the opportunity to evaluate established treatment strategies, thus comparing interventions in its optima forma. Preferably, previous treatment results of the surgical school are published. Publication of these results ascertains quality of the existing surgical school. Also, allocation of the intervention should be a (nearly) random process. In other words, patient referral to the surgeon should be random and does not introduce an indication bias by the referring physician. Generally, trauma patients will receive care from the nearest hospital able to facilitate adequate care. Therefore, treatment allocation for trauma patients usually is determined by the geographic location of the trauma and the assumption of random allocation of intervention can be made. All known (possible) confounders need to be collected and accounted for in the final analysis. If a large effect size is anticipated, observational studies may be sufficient to prove the superiority of one treatment over the other. In this situation, it may be unethical to perform an RCT. On the other hand, small effect sizes are extremely difficult to differentiate from bias in an observational study. If researchers expect a new treatment to offer only minor advantages, such minor effects may be hard to separate from small bias effects possibly present in observational studies. At the same time, conducting a randomized trial to detect a small advantages may not be worthwhile either. Many guideline groups have adopted the GRADE methods, which allow upgrading the credibility of observational research if a large magnitude of an effect was found [39].

Furthermore, researchers should focus on defining objective endpoints that are directly related to the surgical intervention (e.g. mortality, infection, hospital length of stay, non-union, patient reported outcome measured in a standard fashion) in order to obtain valid results and reduce the risk of bias in endpoint assessment. As surgeons and other treatment staff usually are (or have to be) aware of treatment, blinding in surgical trials is often limited to assessment of outcomes. Nevertheless blinded assessment of outcomes (done either by patients or by research staff) increases the validity not only in randomized but also in observational studies. Finally, experienced methodologists and statisticians should be involved from design to execution of the study.

## Future goals for surgical research: towards question-based study designs

Current evidence regarding equivalence of results from different study designs comes from a handful of meta-analyses comparing different treatment strategies (type 1, type 2, and type 3 comparisons). Future studies should aim to replicate these studies restricted to surgical topics and stratified by type of comparison (type 1, 2, and 3) to strengthen current support for complementary use of observational studies. Particularly for type 2 comparisons, it should become common practice in surgery to include both RCTs and observational studies in meta-analysis to enable the full potential of the existing literature in the constant search for the optimal treatment strategy and its associated complications and long-term outcome. Results of the different designs should be regarded as complementary to each other when evaluating surgical interventions.

## Conclusion

We have discussed differences between RCTs and observational studies and differentiate between three types of comparisons of surgical interventions. Observational studies can be of major importance in the field of surgical research. Results of different designs should be regarded as complementary to each other when evaluating surgical interventions; what matters is the quality of the conducted study, not the design per se. Criteria for credible observational research are presented (SPACE-ME) to provide guidance for future observational research of surgical interventions. Surgical research includes various types of comparisons, including comparisons of pharmacological treatment and comparisons of surgical interventions. The potential value of the observational study design differs between the different possible comparisons. We argue that the research question that is being asked, or the comparison that is being made, should guide the discussion about the value of a particular study design.
